# **An** RNA-interference **screen in *Drosophila* to identify ZAD-containing C2H2 zinc finger genes that function in female germ cells**

**DOI:** 10.1093/g3journal/jkaa016

**Published:** 2020-12-07

**Authors:** Laura Shapiro-Kulnane, Oscar Bautista, Helen K Salz

**Affiliations:** Department of Genetics and Genome Sciences, Case Western Reserve University, School of Medicine, 10900 Euclid Ave. Cleveland, OH 44106, USA

**Keywords:** multigene family, reproduction, oogenesis, gametogenesis, Zif, odj, M1PB, CG320020, trem, hang, CG4936, CG17802

## Abstract

The zinc finger-associated domain (ZAD) is present in over 90 C2H2 zinc finger (ZNF) proteins. Despite their abundance, only a few ZAD-ZNF genes have been characterized to date. Here, we systematically analyze the function of 68 ZAD-ZNF genes in *Drosophila* female germ cells by performing an *in vivo* RNA-interference screen. We identified eight ZAD-ZNF genes required for oogenesis, and based on further characterization of the knockdown phenotypes, we uncovered defects broadly consistent with functions in germ cell specification and/or survival, early differentiation, and egg chamber maturation. These results provide a candidate pool for future studies aimed at functionalization of this large but poorly characterized gene family.

## Introduction

In eukaryotes, C2H2 zinc finger (ZNF) proteins are among the most abundant class of DNA-binding proteins (Stubbs *et al.* 2011; Fedotova *et al.* 2017). ZNF proteins are often grouped into families based on the presence of conserved N-terminal domains. In *Drosophila*, over 90 ZNF proteins belong to the zinc finger-associated domain (ZAD) family. The N-terminal ZAD domain can mediate protein–protein interactions, whereas the ZNF domain can confer DNA-binding specificity (Jauch *et al.* 2003). Despite their abundance, the function of only a few ZAD-ZNFs has been examined *in vivo.* These studies suggest essential roles in regulating transcription, silencing transposons, and organizing chromatin architecture during development (Fedotova *et al.* 2017). Interestingly, the ZAD-ZNF family is mostly restricted to *Drosophila* and related species (Chung *et al.* 2002, 2007). Because many of the ZAD-ZNF family members are expressed in ovaries, it has been suggested that the expansion of this gene family was driven by species-specific requirements in the germline (Chung *et al.* 2002, 2007). However, most of the ZAD-ZNF genes remain uncharacterized and their role in the female germline unknown.

In *Drosophila*, each adult ovary is subdivided into 16–20 individual ovarioles, which contain oocytes spanning the range of maturity, from germline stem cell (GSC) to fully mature eggs (Hinnant *et al.* 2020). Two to three GSCs are located at the anterior-most structure of the ovariole, called the germarium. Each GSC divides asymmetrically to produce one daughter cell that retains its GSC identity and a second daughter cell called a cystoblast (CB) that will enter the differentiation pathway. The differentiation program begins with four synchronous incomplete mitotic divisions to give rise to a 16-cell interconnected cyst from which one cell becomes the oocyte and enters meiosis. The other 15 cells, called nurse cells, will provide necessary RNA and protein products to the developing egg. Once the 16-cell cyst is formed, the somatic follicle cells surround the cyst in a monolayer to generate an egg chamber.

In this study, we tested the function of 68 ZAD-ZNF genes in the female germline. Using RNA-interference (RNAi) to knock down expression in germ cells, we identified eight ZAD-ZNF genes whose products are required for egg production. The linear arrangement of oocyte production allows the unequivocal identification of each stage development by location, morphology, and expression of molecular markers. Analysis of the mutant ovaries using these methods uncovered defects consistent with functions in germ cell specification and/or survival, early differentiation, and egg chamber maturation. These results provide a candidate pool for future studies.

## Materials and methods

### Primary germline-specific RNAi screen

We searched Flybase to identify all genes that encode a ZAD domain (ZNF, AD-type, IPR012934; http://www.ebi.ac.uk/interpro/entry/InterPro/IPR012934/). We identified 93 ZAD-ZNF encoding genes (Table 1). Screening was limited to the 68 ZAD-ZNF genes for which germline-optimized TRiP RNAi lines (in VALIUM 20, 21 or 22) were available at the Bloomington Drosophila Stock Center (BDSC stock numbers listed in Table 1). Expression of the UAS-RNAi transgenic lines was driven by *nanos-GAL4* (P{GAL4::VP16-nos.UTR}, BDSC #4937), which is strongly expressed in germ cells. Males from each UAS-RNAi line were crossed to *nanos-GAL4* females at 29°C, and the F1 female progeny tested for egg production.

**Table 1:** List of annotated ZAD-ZFP genes, the TRiP transgenic lines used for the primary screen and a summary of the results

FBgn number	Gene name	Symbol or CG number	Germline-optimized TRiP transgenic lines used listed by BDSC stock #s	Numbers of >RNAi at 29 phenotype
FBgn0026575	Hangover	hang	#41870, #35674, #57791	2/3 No eggs
FBgn0037446	Zinc-finger protein	Zif	#36641	No eggs
FBgn0037621	Motif 1-binding protein	M1BP	#32858, #41937	No eggs
FBgn0038549	–	CG17802	#43233, #57480	No eggs
FBgn0038551	Oddjob	Odj	#62184, #61912	No eggs
FBgn0038767	trade embargo	trem	#40881	No eggs
FBgn0038768	–	CG4936	#64550	No eggs
FBgn0050020	–	CG30020	#58110	No eggs
FBgn0000520	Deformed wings (zw5)	dwg	#35666	Eggs
FBgn0001133	grauzone	grau	#53251, #41940	Eggs
FBgn0001990	weckle	wek	#35680, #57260	Eggs
FBgn0022699	D19B	D19B	#51166	Eggs
FBgn0022935	D19A	D19A	#33371	Eggs
FBgn0024975	–	CG2712	#57418	Eggs
FBgn0025874	Meiotic central spindle	Meics	#50636	Eggs
FBgn0029895	–	CG14441	#67271	Eggs
FBgn0029928	–	CG3032	#57560	Eggs
FBgn0030206	–	CG2889	#77384	Eggs
FBgn0030316	–	CG11695	#57246	Eggs
FBgn0030455	–	CG4318	#51720	Eggs
FBgn0030659	–	CG9215	#57537	Eggs
FBgn0031144	–	CG1529	#60094	Eggs
FBgn0031597	–	CG17612	#57540	Eggs
FBgn0031608	–	CG15435	#65064	Eggs
FBgn0031610	–	CG15436	#57867	Eggs
FBgn0032223	GATAd	GATAd	#33747, #34625, #34640	Eggs
FBgn0032730	–	CG10431	#44470	Eggs
FBgn0033581	–	CG12391	#40847	Eggs
FBgn0034062	–	CG8388	#57545	Eggs
FBgn0034114	–	CG4282	#62445	Eggs
FBgn0034878	pita	pita	#35724, #57732	Eggs
FBgn0035036	–	CG4707	#61167, #58229	Eggs
FBgn0035691	–	CG7386	#62181	Eggs
FBgn0036395	–	CG17361	#57714	Eggs
FBgn0037085	Neuroectoderm-expressed 2	Neu2	#60002	Eggs
FBgn0037584	–	CG7963	#60116	Eggs
FBgn0037617	Numerous disordered muscles	nom	#43551	Eggs
FBgn0037618	ouija board	ouib	#42531	Eggs
FBgn0037619	–	CG8159	#62182	Eggs
FBgn0037620	ranshi	ranshi	#43156, #58206	Eggs
FBgn0037717	–	CG8301	#41643, #62206	Eggs
FBgn0037722	–	CG8319	#61173	Eggs
FBgn0037794	–	CG6254	#57279	Eggs
FBgn0037876	–	CG4820	#60117	Eggs
FBgn0037877	–	CG6689	#67282	Eggs
FBgn0037921	–	CG6808	#35588	Eggs
FBgn0037922	–	CG14711	#57280	Eggs
FBgn0038301	–	CG6654	#62183	Eggs
FBgn0038547	–	CG17803	#60022	Eggs
FBgn0038548	–	CG17806	#77448	Eggs
FBgn0038550		CG17801	#60118	Eggs
FBgn0038765	–	CG4424	#43218, #77900	Eggs
FBgn0038766	–	CG4854	#42833	Eggs
FBgn0039355	–	CG4730	#61169	Eggs
FBgn0039602	–	CG1647	#55292	Eggs
FBgn0040467	Dorsal interacting protein 1	Dlip1	#57717	Eggs
FBgn0042205	–	CG18764	#64554	Eggs
FBgn0050431	–	CG30431	#60023	Eggs
FBgn0051109	–	CG31109	#61878	Eggs
FBgn0051365	–	CG31365	#57450	Eggs
FBgn0051388	–	CG31388	#67288	Eggs
FBgn0051441	–	CG31441	#67285	Eggs
FBgn0051457	–	CG31457	#63588	Eggs
FBgn0067779	debra	dbr	#43222	Eggs
FBgn0260741	–	CG3281	#44555	Eggs
FBgn0263490	Molting defective	mld	#43145, #63020	Eggs
FBgn0264744	–	CG44002	#57463	Eggs
FBgn0285971	piragua	prg	#36631	Eggs
FBgn0014931	–	CG2678	N/A	N/A
FBgn0028647	–	CG11902	N/A	N/A
FBgn0028895	–	CG17328	N/A	N/A
FBgn0030240	–	CG2202	N/A	N/A
FBgn0030314	–	CG11696	N/A	N/A
FBgn0032150	–	CG13123	N/A	N/A
FBgn0032763	–	CG17568	N/A	N/A
FBgn0032814	–	CG10366	N/A	N/A
FBgn0033569	–	CG12942	N/A	N/A
FBgn0034379	–	CG15073	N/A	N/A
FBgn0034643	–	CG10321	N/A	N/A
FBgn0035690	–	CG10274	N/A	N/A
FBgn0035702	–	CG10147	N/A	N/A
FBgn0036294	–	CG10654	N/A	N/A
FBgn0036396	–	CG17359	N/A	N/A
FBgn0037317	–	CG14667	N/A	N/A
FBgn0037920	–	CG14710	N/A	N/A
FBgn0037923	–	CG6813	N/A	N/A
FBgn0037931	–	CG18476	N/A	N/A
FBgn0038418	poils au dos	pad	N/A	N/A
FBgn0039329	–	CG10669	N/A	N/A
FBgn0039860	–	CG1792	N/A	N/A
FBgn0043796	–	CG12219	N/A	N/A
FBgn0260243	Enhancer of variegation 3-9	E(var)3-9	N/A	N/A

The genes are sorted as follows: (1) genes for which germline-specific knockdown resulted in female sterility (no eggs). (2) genes for which germline-specific knockdown did not disrupt female fertility (eggs), and (3) genes for which we were not able to obtain germline-optimized RNAi lines (N/A).

### Immunofluorescence and image analysis

F1 females were raised and aged 3–5 days at 29°C prior to gonad dissection. Ovaries were fixed and stained according to standard procedures with the following primary antibodies: rat anti-HA high affinity (1:500, Roche cat #11867423001, RRID: AB_390919), rat anti-*Drosophila* Vasa (1:100, Developmental Studies Hybridoma Bank, RRID: AB_760351), and mouse anti-*Drosophila* Orb 4H8 (1:50, Developmental Studies Hybridoma Bank, RRID: AB_528418) combined with mouse anti-*Drosophila* Orb 6H4 (1:50, Developmental Studies Hybridoma Bank, RRID: AB_528419). Staining was detected with the following conjugated antibodies: Fluorescein (FITC) AffiniPure Goat anti- rat IgG (H + L) (1:200, Jackson ImmnoResearch Labs, cat #112-095-062, RRID: AB_2338194), or Fluorescein (FITC) AffiniPure Goat Anti-mouse IgG (H + L) (1:200, Jackson ImmnoResearch Labs, cat #115-095-003, RRID: AB_2338589). TO-PRO-3 Iodide monomeric cyanine nucleic acid stain (Thermo Fisher, cat #T3605) was used to stain DNA.

Images were taken on a Leica SP8 confocal with 1024 × 1024 pixel dimensions, a scan speed of 600 Hz, and a frame average of 3. Sequential scanning was done for each channel and three *Z*-stacks were combined for each image. Processed images were compiled with Gnu Image Manipulation Program and Microsoft PowerPoint.

The HA-tagged *bam* transgene, P{bamP-Bam::HA}, was used to report on BAM protein expression (Li *et al.* 2009).

### Data availability statement

*Drosophila* strains are available upon request. The authors affirm that all data necessary for confirming the conclusion of this study are present within the article, figures, and tables.

## Results and discussion

### Germline depletion screen identifies eight ZAD-ZNF genes necessary for oogenesis

In the primary screen, we knocked down the expression of 68 ZAD-ZNF genes by using the germ cell-specific driver *nanos-GAL4* to express the germline-optimized TRiP UAS-RNAi transgenes. Germline knockdown (GLKD) of only eight genes abolished egg production (Table 1). For the remaining 60 genes, knocking down expression in germ cells did not prevent egg production. While the lack of phenotype suggests that the majority of ZAD-ZNF genes are not required intrinsically for germ cell development, we cannot rule out other explanations for the lack of mutant phenotype, such as low RNAi efficiency of the available TRiP lines. Furthermore, our GLKD screen would not have identified genes required in somatic cells that contribute to germ cell development.

### Secondary immunofluorescence screen to characterize ovarian defects

**Figure 1:**
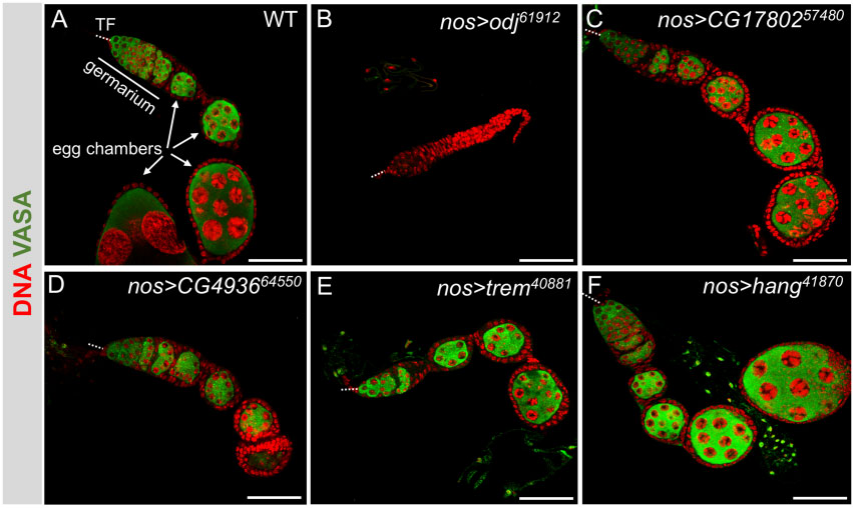
Loss of 8 ZAD-ZNF genes in germ cells disrupt oogenesis. Representative confocal images of control (A) and mutant ovarioles (B–F) stained for Vasa (green) and DNA (red). All images include the somatic terminal filaments (TF, dotted line), the germarium, and early-stage egg chambers (EC). Scale bar 50 µm. Quantification of phenotypes is presented in Figure 2.

**Figure 2:**
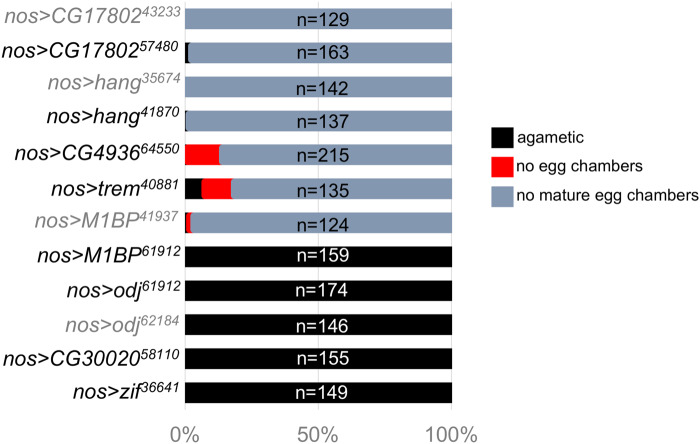
Range of defects observed in ZAD-ZNF GLKD mutant ovaries. Bar graph showing the percentage of VASA stained ovarioles with no germ cells (agametic), no egg chamber formation, or no mature, vitellogenic, egg chambers. Examples of the most prevalent phenotypes are shown in Figure 1.

For the eight genes whose GLKD abolished egg production, we analyzed the ovarian defects by staining for DNA and Vasa, a germ cell-specific marker. For each GLKD experiment, >120 ovarioles were scored individually, as: (1) agametic, defined by the absence of Vasa-positive cells, (2) a failure to form egg chambers, and (3) late defects, in which egg chambers are formed but fail to progress to the larger, more mature stages. The wild-type ovariole has a stereotypical organization with all stages of oogenesis arranged in developmental order from the GSCs housed at the anterior end (the germarium) to the progressively larger, more developed egg chambers (Figure 1A). We found that GLKD of some of the genes gave rise to more than one ovarian defect, and the “per ovariole” analysis, reported in Figure 2, allowed us to quantify the penetrance of each phenotype. Based on this analysis, we conclude that the GLKD phenotypes fall into two general classes: agametic and the absence of the larger, late-stage egg chambers (late defects). Several of the candidate genes were targeted by more than one *UAS-RNAi* line. In the following sections, we limit our description to the line that induced the most severe phenotype.

*Agametic:* We found that >90% of the *Zif*, *odj*, *M1PB*, and *CG320020* knockdown ovarioles had no germ cells (no Vasa-positive cells, see for example Figure 1B). This agametic phenotype suggests a requirement for germ cell specification, and/or survival. A requirement for *odj* was confirmed by the second *UAS-RNAi* line, which recapitulated the agametic phenotype.

**Figure 3:**
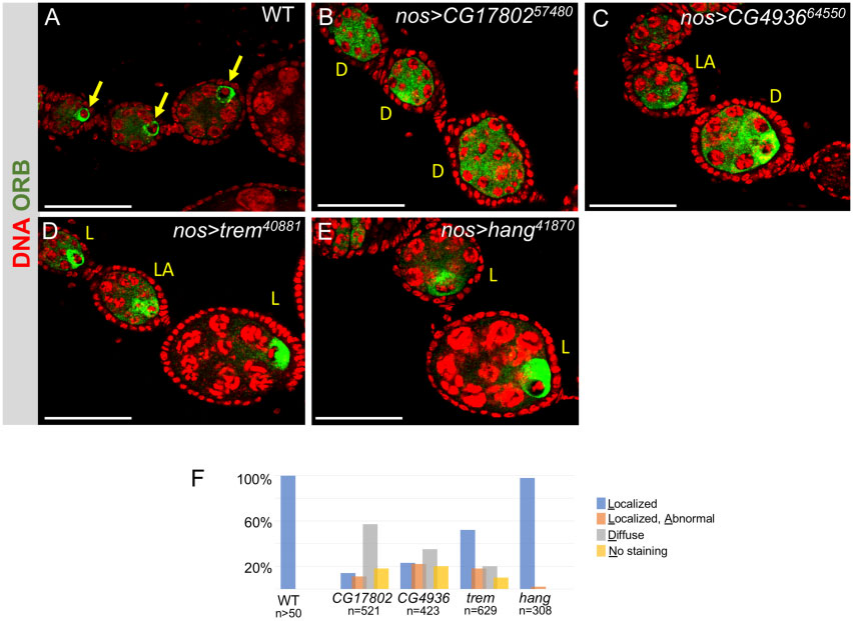
Impact of ZAD-ZNF germ cell-specific knockdowns on oocyte development. Representative confocal images of egg chambers from control (A) and mutant ovarioles (B–E) stained for Orb (green) and DNA (red). In wild type, the Orb protein only accumulates around the presumptive oocyte (arrow). The range of phenotypes observed in mutants is indicated as follows: localized (L), localized abnormal (LA), and diffuse (D). Scale bar = 50 µm. (F) Quantification of mutant egg chamber phenotypes. Two to three egg chambers per ovariole were scored. *N* is the number of individual egg chambers scored.

*Late defects:* We found that >80% of the *trem*, *hang*, *CG4936* and *CG17802* GLKD ovarioles formed only pre-vitellogenic egg chambers (see for example Figure 1, C–F). To better understand the role of these “late defect” genes, we stained the GLKD ovaries with an antibody against Orb, a germline-specific marker for oocyte determination. In the wild-type ovary, the Orb protein concentrates around the cytoplasm of the oocyte (Figure 3A). Major defects were noted in *CG17802*, *CG4936* and *trem GLKD* egg chambers. Orb staining remained diffuse in the majority (57%) of *CG17802 GLKD* egg chambers, suggesting a defect in oocyte specification (Figure 3, B and F). On occasion, we observed Orb protein accumulation in the cytoplasm of one (14%) or two (11%) mutant germ cells, or no staining at all (18%). The Orb staining patterns we observed in *CG4936 GLKD* mutant egg chambers ranged from no staining at all (20%), diffuse staining (35%), localization to more than one germ cell (22%), and localization to a single germ cell (23%; Figure 3, C and 3F). Although some *trem GLKD* egg chambers displayed defects in Orb localization, Orb was appropriately localized in the majority (52%; Figure 3, D and F). Lastly, Orb protein appeared to be appropriately localized to a single cell in the majority (98%) of *hang GLKD* egg chambers, suggesting a later defect in egg chamber growth (Figure 3, E and F).

**Figure 4:**
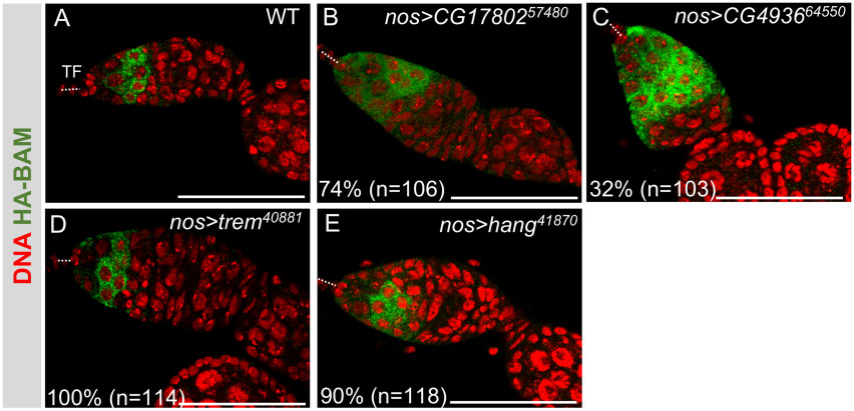
Impact of ZAD-ZNF germ cell-specific knockdowns on expression of the differentiation factor Bam. Representative confocal images of control (A) and mutant ovarioles (B–E) carrying a fully functional copy of a HA-Bam fusion protein, stained for HA (green) and DNA (red). In wild type, Bam protein is only expressed in a few early germ cells. Of note, Bam is not detectable in GSCs, located at the anterior end of the germarium. The location of the somatic terminal filaments (TF) at the anterior end of the germarium is marked by a dashed line. Scale bar = 50 µm. *N* is the number of germaria scored.

To explore whether any of these mutant phenotypes can be explained by defects that occur prior to oocyte specification, we examined the expression of the cytoplasmic differentiation factor Bag of Marbles (Bam). When GSCs divide, the daughter cell that stays at the tip remains a GSC, while the more posterior daughter cell becomes a CB. Each germarium normally contain a single CB, which goes on to divide four times with incomplete cytokinesis, resulting in a 16-cell germline cyst. When we assessed Bam protein expression with a human influenza hemagglutinin (HA)-tagged *bam* transgene (Li *et al.* 2009), we observed that the Bam protein is readily detectable in just a few cells (Figure 4A). Interestingly, we observed an expansion of Bam protein expression in a majority of *CG17802 GLKD* germaria (74%) and in a significant fraction of *CG4936 GLKD* germaria (32%), suggesting defects prior to oocyte specification (Figure 4, B and C). On the other hand, Bam protein accumulation is similar to wild type in *trem* and *hang* GLKD ovaries, suggesting that these genes do not impact the earliest stages of germ cell differentiation (Figure 4, D and E). These data, together with the observation that Orb was appropriately localized in the majority of *trem* and *hang* GLKD egg chambers, suggest that the primary function of *trem* and *hang* is after oocyte specification.

In summary, we have identified eight ZAD-ZNF genes required in germ cells for oogenesis. Several of the ZAD-ZNF genes identified in this study have previously been implicated in germline function, the best characterized of which is *trem.* Trem is a chromatin-associated protein required for the initiation of meiotic double-strand break formation (Lake *et al.* 2011). Although not characterized in detail, *Zif*, *CG4936*, and *hang* were identified in an unbiased RNAi screen for factors required to silence transposons (TE) (Czech *et al.* 2013). However, mutations in genes dedicated to silencing TEs in germ cells induce sterility while maintaining normal ovarian development. Our observations that the loss of *Zif*, *CG4936*, and *hang* disrupts oogenesis suggest a requirement beyond TE silencing.

This analysis also identified ZAD-ZNF genes with no prior reported function in germ cells. For example, we identified a role for the genes encoding the Odj and CG17802 proteins that localize to heterochromatin in somatic cells (Swenson *et al.* 2016; Kasinathan *et al.* 2020). Heterochromatin establishment and maintenance is essential for many cellular processes, including chromosome segregation and genome stability (Janssen *et al.* 2018). A role in germ cells is therefore not entirely unexpected, as mutations in genes that encode crucial heterochromatin functions often disrupt oogenesis (*e.g.*Giauque and Bickel 2016; Teo *et al.* 2018; Smolko *et al.* 2018; Jankovics *et al.* 2018; Casale *et al.* 2019; Benner *et al.* 2019).

We also identified a role for M1BP, a transcription factor that associates with thousands of genes in somatic cells (Li and Gilmour 2013; Zouaz *et al.* 2017; Barthez *et al.* 2020). Similarly, CG30020 is also likely to be transcription factor, as *in vitro* studies have shown that it binds a unique DNA consensus sequence (Krystel and Ayyanathan 2013). Whether CG30020 and M1BP control transcription in a tissue-specific manner remains to be determined.

Overall, the results reported here identify eight members of the large but poorly characterized ZAD-ZNF gene family required in the female germline. The ZAD-ZNF family, which arose in the ancestor of arthropods and vertebrates, expanded to become the most abundant class of transcription factors in *Drosophila* and related species (Chung *et al.* 2002, 2007). Our findings suggest that expansion of the ZAD-ZNF family is related to species-specific requirements in the germline. Future work will test these ideas by a more detailed analysis of these ZAD-ZNF genes in *Drosophila* and other species.
